# In vivo MEMRI characterization of brain metastases using a 3D Look-Locker T1-mapping sequence

**DOI:** 10.1038/srep39449

**Published:** 2016-12-20

**Authors:** Charles R. Castets, Néha Koonjoo, Andreea Hertanu, Pierre Voisin, Jean-Michel Franconi, Sylvain Miraux, Emeline J. Ribot

**Affiliations:** 1 Centre de Résonance Magnétique des Systèmes Biologiques, UMR5536, CNRS/Université de Bordeaux, 146 rue Léo Saignat, 33076 Bordeaux, France

## Abstract

Although MEMRI (Manganese Enhanced MRI) informations were obtained on primary tumors in small animals, MEMRI data on metastases are lacking. Thus, our goal was to determine if 3D Look-Locker T1 mapping was an efficient method to evaluate Mn ions transport in brain metastases *in vivo*. The high spatial resolution in 3D (156 × 156 × 218 μm) of the sequence enabled to detect metastases of 0.3 mm^3^. In parallel, the T1 quantitation enabled to distinguish three populations of MDA-MB-231 derived brain metastases after MnCl2 intravenous injection: one with a healthy blood-tumor barrier that did not internalize Mn^2+^ ions, and two others, which T1 shortened drastically by 54.2% or 24%. Subsequent scans of the mice, enabled by the fast acquisition (23 min), demonstrated that these T1 reached back their pre-injection values in 24 h. Contrarily to metastases, the T1 of U87-MG glioma remained 26.2% shorter for one week. *In vitro* results supported the involvement of the Transient Receptor Potential channels and the Calcium-Sensing Receptor in the uptake and efflux of Mn^2+^ ions, respectively. This study highlights the ability of the 3D Look-Locker T1 mapping sequence to study heterogeneities (i) amongst brain metastases and (ii) between metastases and glioma regarding Mn transport.

Calcium is a ubiquitous second messenger that is necessary for tremendous activities especially in tumor cells. Roderick *et al*. and Prevarskaya *et al*. mentioned that the remodeling of calcium transport could be defined as a new cancer hallmark^1^,^2^. Indeed, calcium ions are believed to have important roles in tumor cell proliferation and invasiveness^3^,^4^. Consequently, detecting and quantifying the calcium pathways into cancer cells might increase the accuracy of diagnoses, and open the door to innovative therapy strategies.

One suitable technique that would allow the study of calcium pathways in intact living organisms along with highly resolved imaging is the Manganese-enhanced MRI (MEMRI) approach. MEMRI is a technique that uses manganese ions (Mn^2+^) as a positive MRI contrast agent. These ions are analogs to calcium ions, consequently they get internalized by cells through the same transport systems, enabling therefore the *in vivo* detection and location to where these two ions are accumulating. MEMRI has already been used to detect the neuro-architecture in rodents^5^,^6^,^7^,^8^,^9^,^10^,^11^, to track the neuronal activation in mice^12^ and to evaluate the extent of myocardial infarctions^13^,^14^,^15^,^16^. Furthermore, several studies already demonstrated that MEMRI can be employed for oncology, to detect tumors over several stages^17^. Some have noted a difference in manganese uptake between malignant and normal cells^18^. Baio *et al*.^19^ detected a high accumulation of Mn^2+^ ions in breast cancer cells expressing high levels of the Calcium-Sensing Receptor (CaSR). Furthermore, a previous study has clearly demonstrated that Mn^2+^ uptake of three different cell lines was correlated to their proliferation rates *in vitro*^20^. This study has been proven to be useful to evaluate proliferation of tumor cells *in vivo* after radiotherapy^21^.

Although the detection of Mn^2+^ ions *in vivo* brings anatomical information, it is also necessary to quantify the uptake, efflux and residual time of the contrast agent in order to follow longitudinally cancer cell activity and evaluate the efficiencies of therapeutic approaches. To do so, measuring the effect of Mn^2+^ ions on the longitudinal (T1) relaxation time seems to be a straightforward procedure as, at first, the T1 relaxation process is not affected by the heterogeneities in the magnetic field and second, the shortening of T1 relaxation time constant is dependent on the variation of the Mn^2+^ ion concentration. Several studies have measured the T1 in tumors after the injection of MnCl2. The limitation of these studies was the acquisitions of T1 maps in 2D on a limited number of slices through the tumors^21^,^22^,^23^. Only a few studies acquired T1 maps in 3D, with the main drawback of long acquisition times: from 40 min^24^ to ~2 h^25^. In addition, all these published works used the Variable Flip Angle technique, that largely overestimates the T1 values^26^. Recently, Castets *et al*. developed a 3D T1 mapping sequence combining the Look-Locker module with a spiral sampling that lasted only 12 min with a spatial resolution of 208 × 208 × 312 μm on the mouse’s heart^13^. This type of sequence would be very useful to localize precisely Mn^2+^ ions within tumors, to follow the kinetics of Mn^2+^ ions in the tumors and finally to detect small early-growing metastases. Indeed, even though MEMRI has been used often to image primary tumors, there is a lack of MEMRI information on metastases. To date, these secondary tumors are getting tremendous interest due to the drop of patients’ survival rate. For example, the 5-year survival rate for breast cancer patients is about 25% as from when the metastatic disease is diagnosed^27^. This rate is affected by the increasing incidence of brain metastases^28^.

Consequently, the goal of our study was to determine if 3D Look-Locker T1 mapping could be an efficient and precise method to evaluate Mn ions transport in brain metastases *in vivo*. 3D T1 maps were acquired on human breast cancer-derived brain metastases in mice, and the uptake and efflux of Mn^2+^ ions were compared between the metastases. Secondly, this protocol was applied on aggressive primary brain tumors implanted orthotopically so as to compare, in the brain environment, their behaviors with those of the metastases. *In vitro* experiments were then performed in an attempt to determine the implication of two main calcium-transporting proteins (the Transient Receptor Potential (TRP) channels and the Calcium-Sensing Receptor (CaSR)) in the *in vivo* MEMRI quantification.

## Results

### *In vivo* 3D spiral Look-Locker T1 maps of metastases

**Figure 1:**
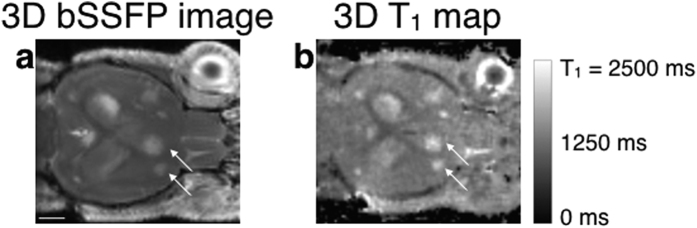
3D bSSFP and 3D T1 map of the brain of a mouse bearing metastases. Metastases detected on the bSSFP image (**a**) as hyper-intense areas are also detected on the T1 map before injection (**b**), due to their longer T1 than healthy brain. The two metastases pointed out (arrows) have drastically different volumes but similar T1 values. The scale bar represents 2 mm.

Figure 1 shows non enhanced 3D bSSFP images and the corresponding 3D T1 maps of several brain metastases. All the metastases appeared with a bright signal on both sets of images. On the bSSFP images, the hyper signal was due to the T2/T1 contrast of the sequence and the watery composition of the metastases which also accounted for longer T1 values on the 3D T1 maps. Supplementary Figure 1 enables to appreciate the high spatial resolution of the T1 maps, in the axial, coronal and sagittal orientations. A metastasis of less than 0.3 mm^3^ can thus be easily detected (arrow in Supplementary Figure 1b). The different structures of the brain, like the corpus callosum or the ventricles, can also be easily identified. Similar amounts of metastases were detected on both the bSSFP images and the 3D T1 maps. A heterogeneity in the T1 values was observed amongst the metastases within a mouse. Some had T1 close to the one of the healthy brain (1276.5 ± 73.5 ms) whereas some had longer T1 (appearing in bright on the T1 maps). Consequently, the mean T1 value was measured to be 1564.4 ms with a large standard deviation of 267.8 ms. It is important to note that the T1 values were not in agreement with the metastasis volumes, as demonstrated with two metastases (Fig. 1 arrows) with similar T1 values (1866 ms and 1769 ms) but with different volumes (0.9 mm^3^ and 0.3 mm^3^, respectively).

### *In vivo* MEMRI of metastases using the 3D spiral Look-Locker T1 maps

**Figure 2:**
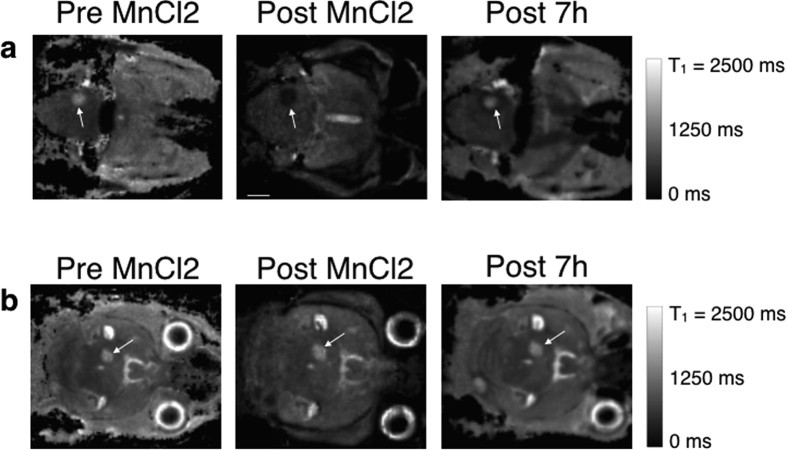
3D T1 maps of a mouse brain with metastases over time after the MnCl2 injection. Two slices showing two metastases (arrows) with similar volumes are shown. The metastasis in (**a**) took up Mn^2+^ ions, whereas the T1 of the metastasis pointed out in (**b**) remained stable over time. The scale bar represents 2 mm.

**Figure 3:**
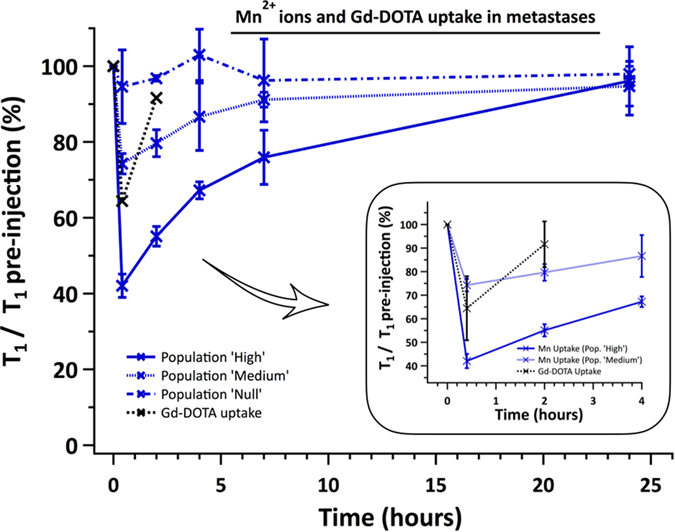
Graph showing the normalized T1 values in metastases over time after the injection of MnCl2 or Gd-DOTA. The T1 post-injection were normalized to the T1 pre-injection. Three significantly different populations of metastases can be distinguished: one with an intact BTB (population « Null », dash-dot blue line), one which large (population « High », plain blue line) or moderate (population « Medium », dashed blue line) T1 decrease. The T1 values of populations « High » and « Medium » remained significantly different until 24 h post-injection (p < 0.01). These two latter populations took up Gd-DOTA, but couldn’t be distinguished (insert, dashed black line). The T1 values reached back their pre-contrast values 2 h or 24 h post-injection, when Gd-DOTA or MnCl2 was injected, respectively.

After an intravenous injection of MnCl2, 3D T1 maps were acquired. Several metastases had their T1 that dropped massively (by 54.2% ± 9.2) demonstrating that Mn^2+^ ions accumulated within the tumors (called Population « High » thereafter, Figs 2a and 3). Two other metastasis populations were found: a similar number of metastases took up Mn^2+^ ions but in a significant lesser extent (p < 0.01) than the metastases studied above (T1 decreased by 24% ± 5, called Population « Medium », Fig. 3) and some metastases didn’t have any significant change in their contrasts with the brain (called Population « Null », Figs 2b and 3). Populations « High » and « Medium » each represented around 36% of all the metastases studied. The metastases in Population « Null » were less numerous (29% of the total amount of metastases analyzed). Consequently, a wide range of T1 values were measured immediately after injection: from 622 ms to 1361 ms.

There was no correlation between the pre-contrast T1 and the intensity of T1 shortening after MnCl2 injection. Indeed, within the High population, metastases had pre-contrast T1 values varying from 970 ms to 2306 ms.

The volume of the metastasis was not related to the ability to internalize Mn^2+^ ions. Indeed, two metastases of similar volumes (1.2 mm^3^ and 1 mm^3^ each) had their T1 values very different right after the injection (739.1 ms and 1310 ms, respectively, Fig. 2 arrows).

### *In vivo* MEMRI of primary glioma tumors

**Figure 4:**
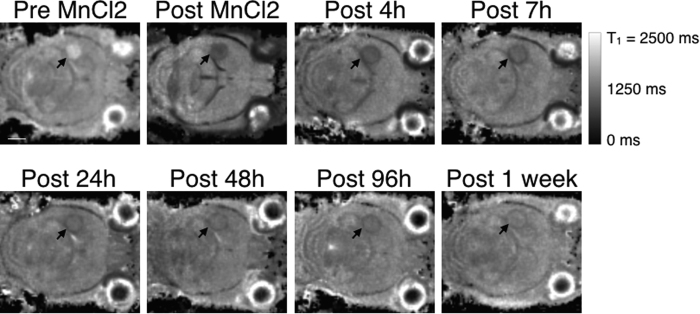
3D T1 maps of the brain of a glioma-bearing mouse over time after the injection of MnCl2. One slice from the 3D maps is shown at different time points: before, immediately after, 4 h, 7 h, 24 h, 48 h, 96 h and 1 week post-injection of MnCl2. The arrow points out the glioma. The scale bar represents 2 mm.

The 3D T1 maps showed that 7 days after implantation, the mean T1 value of glioma was 1734 ± 107 ms (Fig. 4). Immediately after MnCl2 injection, a significant decrease of 39% ± 8.8 in T1 value was measured in the tumor (p < 0.02).

It was interesting to observe that a noticeable rim was detected 4 hours after the injection of MnCl2. The rim had a 16.4% ± 3.1 shorter T1 than the core of the glioma. The rim was detected for the entire week post-injection. Its T1 was 21.4% ± 2.3, 20% ± 0.8, 24.3% ± 0.7, 20.6% ± 3.1 and 8.7% ± 2.9 shorter than the core at 8 h, 24 h, 48 h, 96 h and 1 week post-injection, respectively.

**Figure 5:**
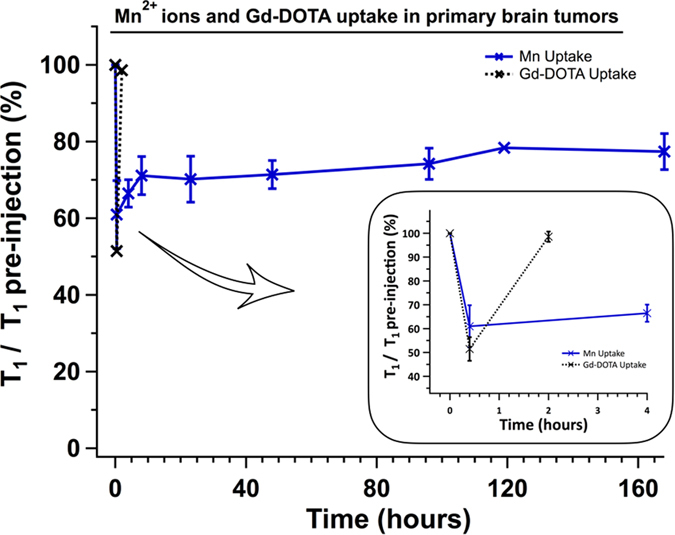
Graph showing the normalized T1 values in glioma over time after the injection of MnCl2 or Gd-DOTA. The T1 post-injection values were normalized to the T1 pre-injection. No heterogeneity in between glioma after MnCl2 injection was observed (plain blue line). Contrarily to a fast clearance of Gd-DOTA (insert, dashed black line), Mn^2+^ ions were still present in the glioma after 1 week (p < 0.02).

The clearance of the Mn^2+^ ions was very slow, as demonstrated on the kinetic curves, where the T1 values didn’t attain the pre-injection values after 1 week (Fig. 5). Indeed, at one week post-injection, the T1 value of the primary tumors recovered at 77.4% ± 4.7 of its pre-contrast value (p < 0.02).

In comparison, the T1 of the healthy brain cortex did not shortened more than 7% in 1 week (data not shown).

### MEMRI versus Gd-DOTA-enhanced MRI of metastases and glioma using the 3D spiral Look-Locker T1 maps

**Figure 6:**
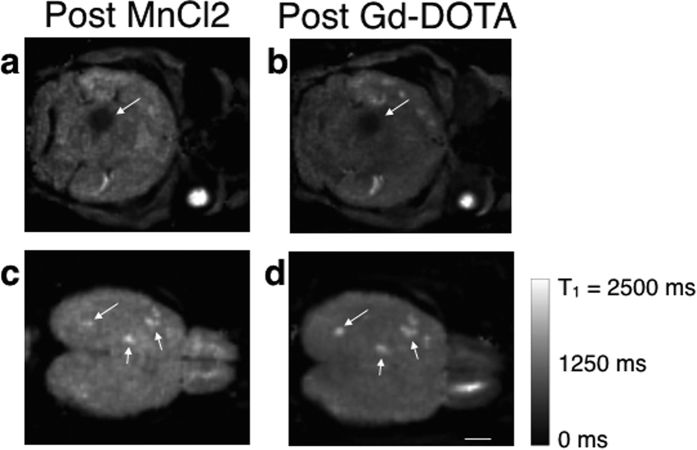
3D T1 maps of mouse brains with metastases, immediately after the injection of MnCl2 or Gd-DOTA. The metastases that internalized Mn^2+^ ions (arrow in **a**) were the ones that had a leaky vasculature (as shown by the short T1 value after Gd-DOTA injection in **b**). On the contrary, metastases with intact BTB didn’t internalize Mn^2+^ or Gd-DOTA (arrows in **c** and **d**). The scale bar represents 2 mm.

The commonly-used Gd-DOTA contrast agent to detect tumors was then intravenously injected in the mice. As shown in Fig. 6b, Gd-DOTA leaked into some metastases (inducing a 36% drop of their T1 values, p < 0.01) as demonstrated by the hypo-intense signal on the acquired 3D T1 maps post injection. Comparably with MEMRI obtained right after the injection of MnCl2, only those metastases with a leaky vasculature could internalize Mn^2+^ ions. Metastases in Fig. 6c,d did not internalize either Mn^2+^ or Gd-DOTA. It is important to note that there was no correlation between the T1 values of the metastases before MnCl2 injection and the permeability of the blood-tumor barrier (BTB). Indeed, two metastases with similar T1 before MnCl2 injection (1252 ms and 1282 ms) belonged to the High and Null populations after injection, respectively.

Contrarily to the T1 of metastases that retrieved their initial values 24 hours post MnCl2 injection, the clearance of Gd-DOTA was faster: the T1 values of the metastases returned back to 91.7% ± 9.7 of their pre-injection values as soon as 2 hours post-injection (Fig. 3).

A similar pattern was measured in the glioma. Following Gd-DOTA injection, the T1 value in the glioma dropped by 48.6% ± 5 (p < 0.05), and returned to its initial value within 2 hours after injection (Fig. 5).

### *In vitro* MEMRI to study influx and efflux of Mn^2+^ ions in cancer cells

In order to evaluate the main route of Mn^2+^ ion uptake by the cancer cells, inhibition of the Transient Receptor Potential (TRP) channels using the broad spectrum blocker SKF-96365^29^ was performed. This treatment induced a large increase in T1 values (around 50%) of both cancer cell lines as compared to untreated Mn^2+^-labeled cells (Table 1). Decreasing SKF-96365 concentrations reduced the T1 lengthening of both cell lines as a dose-dependent effect (Supplementary Figure 2a). On the contrary, incubating the cells with concentrations higher than 50 μM started to generate cytoxicity.

Treatments with Verapamil were also performed in order to evaluate the role of the Calcium-Sensing Receptor (CaSR) in the release of Mn^2+^ ions out of the cancer cells^19^. This treatment enabled to retain Mn^2+^ ions into the metastatic cells, shown by a 34.1% T1 value shortening (Table 1). On the contrary, no significant change on the T1 value of the Mn^2+^-labeled U87-MG cells was measured. This absence of effect was observed even for increasing concentrations of Verapamil (Supplementary Figure 2b).

## Discussion

This paper demonstrates for the first time that the MEMRI technique can be used to detect and study manganese transport in brain metastases.

The 3D T1 mapping protocol developed recently^13^ was used to quantify the T1 variations of brain metastases induced by the presence of Mn^2+^ ions *in vivo*. This sequence not only enabled the detection of Mn^2+^ ions like usual 3D T1-weighted MR sequences, but also, enabled to quantify the Mn^2+^ effects on T1 shortening over time. This is an asset when comparisons between tumors are necessary to evaluate the efficiency of a treatment. The high spatial resolution of the maps (156 × 156 × 218 μm) enabled to study metastases of less than 0.3 mm^3^. Furthermore, as demonstrated by Castets *et al*., this sequence is robust and enables T1 measurements with high precision *in vivo*, due to the recording of 23 points along the longitudinal relaxation^13^. Indeed, most studies limit their T1 calculations with 3–5 points^24^,^25^,^30^, decreasing the accuracy of the T1 values. In addition, the 3D T1 maps were acquired in less than 23 minutes with a high spatial resolution in the three dimensions. This is a real advantage compared to 2D mapping sequences for measuring T1 of small structures like metastases, without partial volume effects. In addition, as the magnetization doesn’t reach back its equilibrium compared to the standard Look-Locker sequences, the TR could be shortened to consequently reduce acquisition time. Chuang *et al*. developed a fast 3D T1-mapping sequence using a Look-Locker module and EPI encoding^31^. In that study, the 3D T1 maps of a rat brain were obtained in 115 min for similar spatial resolutions to the current study. The spiral encoding used here enables to further accelerate acquisition time, to efficiently follow the evolution of a time-resolved biological process in small structures like metastases. Another advantage of this technique is its high sensitivity. Indeed, the current method enabled to detect two degrees of Mn^2+^ uptake (amongst the metastases) and two rates of Mn release (between the metastases and the glioma).

Mn^2+^ ions can be used as an intracellular T1 contrast agent. However, as previously demonstrated by Hee Lee^9^, when intravenously injected, Mn^2+^ ions get into the brain through structures lacking a blood-brain barrier (BBB) like the pituitary gland. As a matter of fact, only metastases with leaky Blood-Tumor Barrier (BTB) were able to uptake Mn^2+^ ions. Similar results were obtained when Gd-DTPA was intravenously injected into brain metastases-bearing mice^32^. However, Gd-DTPA remains in the extracellular compartment, thus limiting its application to cell activity studies. Conversely, Mn^2+^ ions enabled to detect the BBB permeability and simultaneously evaluate calcium transport in metastases and tumors.

The 3D T1 maps demonstrated heterogeneities in the uptake of Mn^2+^ ions in metastases. This might be due to the fact that metastases started to appear at different days post-injection, as already observed by Perera *et al*.^33^. Consequently, several metastasis grades might have been present within the same mouse. The High and Medium populations detected after MnCl2 injection could not be distinguished when Gd-DOTA was injected. Indeed there was no difference in Gd-DOTA uptake amongst the metastases with leaky BTB, demonstrating that the permeability of the vasculature amongst the metastases was high enough to not limit the diffusion of Gd-DOTA (500 Da) and thus of Mn ions (55 Da). This is in agreement with a previous study that showed no correlation between the density and the permeability of the blood vessels present in MDA-MB-231Br metastases^34^. Consequently, these differences measured amongst metastases can further involve the activity and/or expression of calcium pumps. As expected, TRP channels have an essential role in the Mn^2+^ ions uptake for both cell lines. This is in agreement with previous studies that show the role of TRP channels in the proliferation of these tumor cells^35^,^36^.

Concerning the efflux of Mn^2+^ ions from the cancer cells, the *in vivo* measurements of T1 values demonstrated that there was a major difference between the glioma and the metastatic cells. Indeed, contrarily to a rapid clearance from the metastases (less than 24 hours), Mn^2+^ ions remained into the primary tumor cells for a week. In these glioma, an intratumoral heterogeneity was detected with T1 values shorter at the rim compared to the core. This observation is in agreement with a previous study that detected high angiogenic cell activities at the periphery of these tumors^37^. As the T1 values of the healthy cortex did not vary significantly after the injection of MnCl2 (data not shown), the spread of Mn^2+^ ions from healthy parts of the brain towards the glioma that could occur over time can be neglected.

The difference in clearance between brain metastases and glioma, might thus be explained by the activity of the Calcium-Sensing Receptors (CaSR). The measurements of the T1 values demonstrated a clear involvement of the CaSR in the efflux pathways of Mn^2+^ ions from the metastatic cells, but not in the glioma cells. These results are in agreement with previous studies demonstrating high expression of CaSR into the current cell line^38^ and playing an important role into the vicious cycle of breast metastases in bones^39^. Other potential factors may affect the long Mn^2+^ clearance observed in the brain tumors, like the binding of Mn^2+^ ions to the glutamine synthetase expressed in astrocytes^40^.

One limitation of the current study is the inability to evaluate, through the T1 measurements, the effective contribution of intracellular Mn^2+^ ions versus Mn^2+^ ions complexed to extracellular molecules in the T1 values. Gianolio *et al*. determined that 64% of the MR signal was due to intracellular Mn ions in melanoma-bearing mice^41^. In our case, we can also expect a contribution of extracellular Mn ions in the T1 values that we measured. However, as the volumes of the metastases were not correlated to the T1 shortenings and to the permeability of the BTB, the involvement of the extracellular space in the sequestration of Mn ions might be negligible. Consequently, T1 comparisons between metastases are still relevant.

Another limitation of the current study is the inability to inject calcium transport inhibitors *in vivo*, in order to confirm the implications of TRP channels and CaSR in the Mn2+ ions uptake and release in the metastases. Stereotaxic injections in every metastases seem impractical. On one hand, one stereotaxic injection of the drugs in the brain would not ensure similar doses reaching the metastases. On the other hand, systemic administrations of the drugs would necessitate successive injections to maintain calcium pumps/channels inhibitions for several hours, that might affect the health of the animals, especially by their actions in organs like the heart.

In this study, only brain metastases were studied. Performing MEMRI experiments on hepatic or renal metastases might be hampered by the high Mn uptake from these organs participating in the clearance of the contrast agent^42^.

Finally, the last limitations come from the use of spiral encoding which is very challenging, especially because of the complex reconstruction process and the sensitivity to T2* decay^13^.

In conclusion, this study showed for the first time the application of MEMRI on brain metastases. Due to the high spatial and temporal resolutions and the sensitivity of the T1 maps, the results clearly demonstrated that Mn^2+^ ions can be used to detect the leakiness of the tumor vasculature and at the same time to detect dysregulations of manganese transport *in vivo*. The results of the current work should give some insights for further investigations on the mechanisms of Mn^2+^ distribution in brain metastases to develop new therapeutic strategies against calcium transport in metastases.

## Materials and Methods

### Magnet and gradient system

All experiments were performed on a 7 Tesla Bruker Biospec system (Ettlingen, Germany) equipped with a gradient system capable of 660 mT/m maximum strength and 110 μs rise time. A volume resonator (75.4 mm inner diameter, active length 70 mm) operating in quadrature mode was used for excitation and a 4-element (2 × 2) phased array surface coil (outer dimensions of one coil element: 12 × 16 mm^2^, total outer dimensions: 26 × 21 mm^2^) was used for signal reception.

### Cell culture

The human breast cancer cell line (MDA-MB-231Br) and the U87-MG human glioma cells were cultured in DMEM (Dulbecco’s Modified Eagle’s Medium, Invitrogen) containing 10% FBS (fetal bovine serum) at 37 °C and 5% CO_2_.

### Animal models

All experimental procedures were approved by the Animal Care and Use Institutional ethics committee of Bordeaux, France (Comité d’éthique pour l’Expérimentation Animale Bordeaux CEEA50 - approval n°5012032-A) and were performed in accordance with relevant guidelines and regulations.

#### Injection of brain metastatic cells

Female nu/nu mice (N = 6; 8 weeks old; weighing 20–25 g; Charles River Laboratories, L’Arbresle, France) were injected into the left ventricle of the beating heart with 100 μL suspension of 175,000 MDA-MB-231Br cells. Mice were scanned at different time points (at least 21 days after injection).

#### Implantation of primary brain tumors

The U87-MG cells were implanted in female nu/nu mice (N = 6, 8 weeks old, weighing 20–25 g; Charles River Laboratories, L’Arbresle, France) by stereotaxic injection into the striatum, as previously explained^43^. The mice were scanned 7, 8, 9 and 11 days after tumor implantation.

### *In vivo* imaging

In order to detect the brain metastases, a balanced Steady State Free Precession (bSSFP) sequence was first acquired using the following parameters: TE/TR = 2/4 ms; flip angle (FA) = 35°; FOV: 20 × 20 × 14; matrix: 128 × 128 × 64; Bandwidth: 586 Hz/pixel; Number of excitation = 4; 4 offset frequencies; total acquisition time = 9 min 20 s. This sequence was used as a gold standard for brain metastasis detection^33^.

The sequence that was used to obtain 3D T1 maps was based on a stack-of-spiral Look-Locker sequence, as previously described by Castets *et al*.^13^. The following imaging parameters were used: TR/TE = 7.9/1 ms; excitation pulse: cardinal sine, 1 ms; FA = 10°; inversion pulse: gauss, 1 ms; matrix: 128 × 128 × 64; FOV: 20 × 20 × 14 mm; spatial resolution 156 × 156 × 218 μm; Bandwidth: 341 Hz/pixel; points per spiral readout: 878; readout time: 2.9 ms; 12 interleaves per stack of spirals; 64 stacks per k-space; acquisition time: 22 min. Twenty three 3D k-spaces were acquired at 23 inversion times, starting at 7.3 ms and separated by 142 ms.

T1 maps were acquired before and after an intravenous injection of 60 μL of 100 mM of MnCl2. The injection was performed at a rate of 2 μL/min. Neurotoxicity caused by manganese can be an issue when using it as a contrast agent in animal studies. It has been shown that doses until 175 mg/kg injected intravenously do not induce toxicity^9^. In this work, a low dose of manganese (16.5 mg/kg) was injected.

Mice were anesthetized with isoflurane (1.5–2% in air). The animals were positioned within the magnet with the head placed at the center of the NMR coil. A monitoring system of physiologic parameter (SA Instruments, Inc, Stony Brook, NY, USA) enabled the visualization of the respiratory cycle. A T1 map was acquired before the injection, followed by subsequent T1 maps acquisitions at different time points: immediately after injection, at 4, 7, 24, 48, 96 hours and 1 week after injection for primary tumors and immediately after injection, 4, 7 and 24 hours after injection for metastases.

Gd-DOTA (DOTAREM, Guerbet) was also injected intravenously through a rapid bolus at a dose of 100 mM via the tail vein. This contrast agent was injected 24 h prior the injection of MnCl2. 3D T1 maps were also acquired (with the same sequence as described above) before injection, immediately and 2 hours after injection.

### *In vitro* experiments

Cells from the two cell lines were allowed to adhere in 6 cm-petri dishes for 3 days. Then, they were incubated with culture media containing either 100 μM of Verapamil (ab146680, Abcam) during 1 hour or 50 μM of SKF-96365 (Calbiochem, France) during 1 hour. Then, MnCl2 (1 mM) was added into the medium for another 1 hour. Control cells were incubated only with MnCl2 (1 mM) for 1 hour. After 3 washes using the Roswell Park Memorial Institute medium (RPMI, Invitrogen), the cells were collected and spinned twice at 1000 rpm for 6 min. The pellets were then scanned at 7 T using the same MR sequence as the *in vivo* experiments (N = 16 experiments for each cell line).

### Data analysis

The 3D T1 maps were reconstructed using an optimized equation for the sequence used in a previous study^13^ and implemented with Matlab (MathWorks, Natick, MA, USA).

Regions Of Interest (ROI) were manually drawn within 51 metastases (41 metastases from mice used only for the MEMRI experiments, 10 from mice injected with Gd-DOTA and subsequently MnCl2) and 6 glioma (3 glioma from mice used only for the MEMRI experiments, 3 from mice injected with Gd-DOTA and subsequently MnCl2). The T1 was measured as a mean of the T1 values of each voxel within a ROI.

In parallel, the volumes of all the metastases and the glioma were measured in ROIs manually drawn on every slide of the T1 maps where the metastases could be detected using Amira software (TGS, San Diego, CA, USA).

For the *in vitro* experiments, the T1 of SKF-96365-treated Mn-labeled cells was normalized to the T1 of Mn-labeled cells. Similarly, the T1 of Verapamil-treated Mn-labeled cells was normalized to the T1 of Mn-labeled cells. The percentages of T1 lengthening or shortening between treated and untreated Mn-labeled cells are shown in Table 1 and in Supplementary Figure 2.

### Statistical analysis

The T1 values ± Standard Deviations were compared using the paired Student’s t-test. P < 0.05 was considered a significant difference.

## Additional Information

**How to cite this article**: Castets, C. R. *et al*. In vivo MEMRI characterization of brain metastases using a 3D Look-Locker T1-mapping sequence. *Sci. Rep.* **6**, 39449; doi: 10.1038/srep39449 (2016).

**Publisher's note:** Springer Nature remains neutral with regard to jurisdictional claims in published maps and institutional affiliations.

## Supplementary Material

Supplementary Figures

## Figures and Tables

**Table 1:** Percentages of lengthening or shortening on T1 values of the two different cell lines after the treatments with SKF-96365 or Verapamil, respectively.

Treatments	MDA-MB-231Br metastatic cells	U87-MG glioma cells
**SKF-96365**	**↑** 44% ± 3.5	**↑** 49% ± 8
**Verapamil**	**↓** 34.1% ± 8.3	2.7% ± 4.8

The T1 of MDA-MB-231Br cells and U87-MG cells at baseline were 2202 ± 78.9 ms and 2043.5 ± 138.2 ms, respectively.
