# Supplementary material for: In vivo MEMRI characterization of brain metastases using a 3D Look-Locker T1-mapping sequence
Source: Sci Rep. 2016 Dec 20;6:39449. doi: 10.1038/srep39449 (PMC5171659; doi:10.1038/srep39449)
Supplement: Supplementary Figures [file srep39449-s1.pdf]

In vivo MEMRI characterization of brain metastases using a 3D Look-Locker T1-mapping sequence

CR Castets, N Koonjoo, A Hertanu, P Voisin, JM Franconi, S Miraux, EJ Ribot

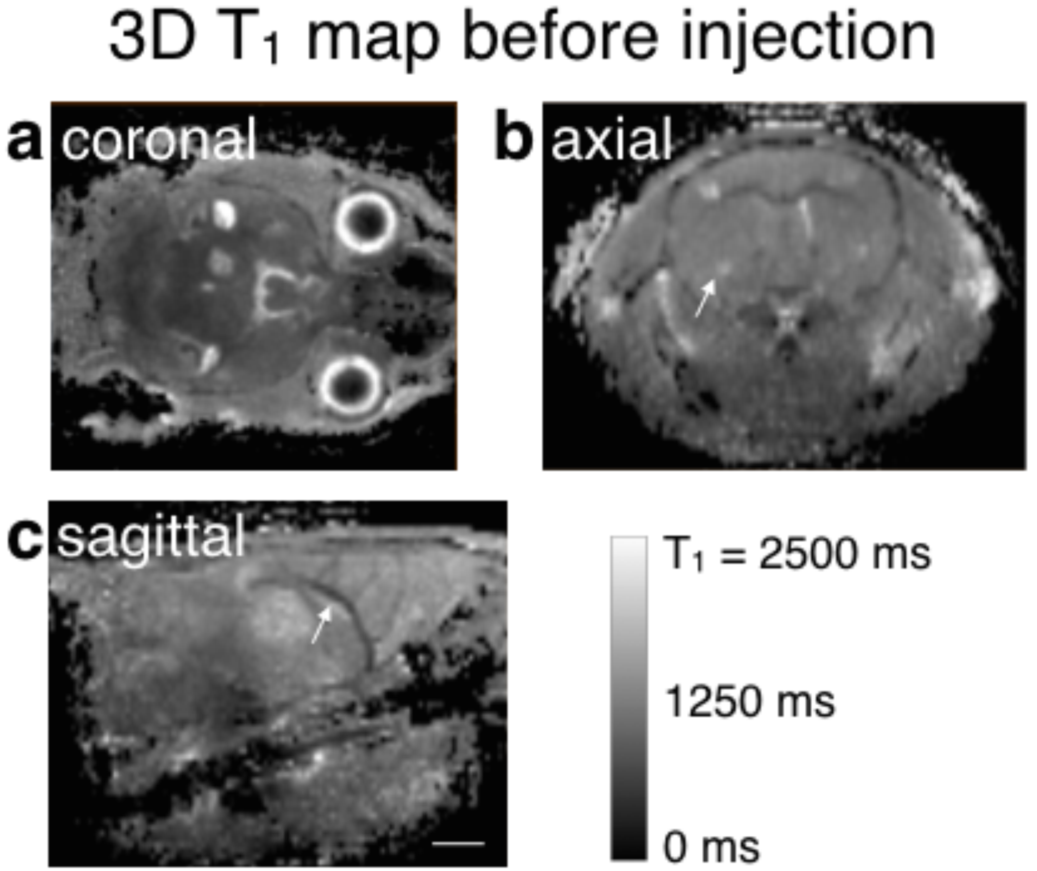

Supplementary Figure 1: Representative images in the three orientations from a 3D T1 map of the brain of a metastases-bearing mouse. Three slices (coronal (a), axial (b) and sagittal (c) orientations) are shown. The high spatial resolution of the maps can be appreciated through the detection of a $0.3mm^3$ metastasis (arrow in b) and the brain vasculature (arrow in c). The scale bar represents 2 mm.

In vivo MEMRI characterization of brain metastases using a 3D Look-Locker T1-mapping sequence

CR Castets, N Koonjoo, A Hertanu, P Voisin, JM Franconi, S Miraux, EJ Ribot

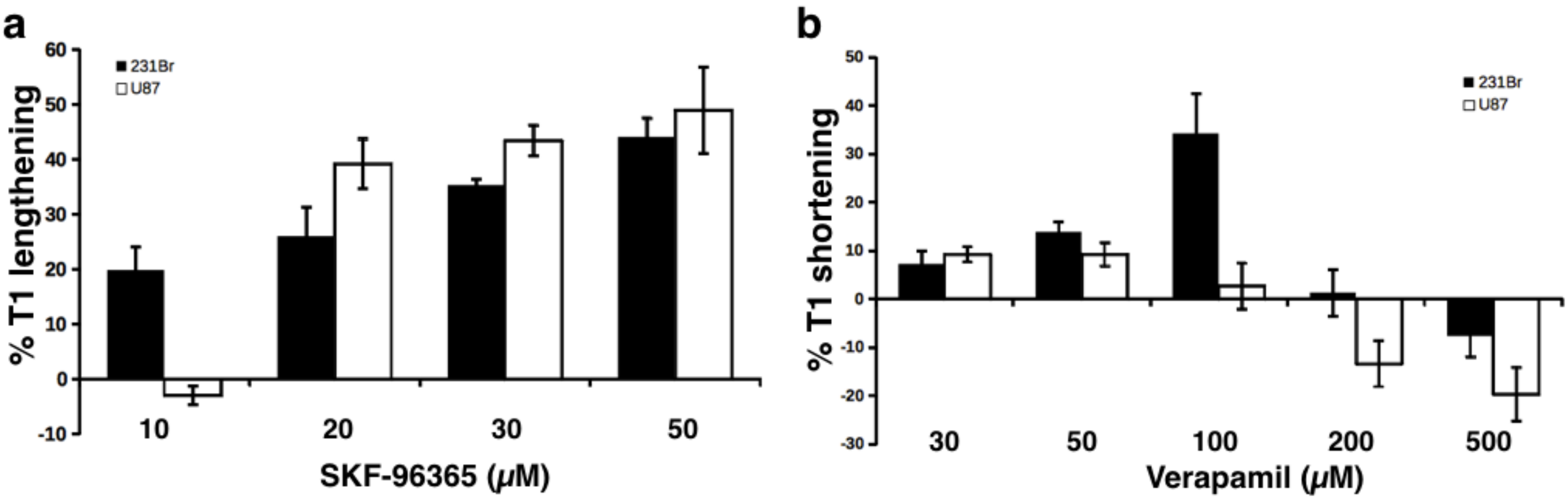

Supplementary Figure 2: Dose-dependent effects of increasing concentrations of SKF-96365 (a) or Verapamil (b) on T1 lengthening or shortening, respectively. As the concentration of SKF-96365 increased, the T1 of the Mn-labeled 231Br cells was lengthening. Similar results were obtained with U87 cells. A concentration above 50μM altered the phenotypic behavior of the cells, demonstrating a cytotoxicity.

The T1 of the Mn-labeled U87 cells was not affected by Verapamil up to a concentration of 200μM, whereas 100μM Verapamil significantly shortened the T1 of Mn-labeled 231Br cells. This confirms that Mn ions release involve the CaSR only in the metastatic cells.

Increasing the concentrations of Verapamil to 500μM induced a contrary effect: the T1 of both cell lines got longer. This might be due to the large spectrum of the Verapamil action. Consequently, through its interaction with voltage-dependent calcium channels, Mn ions entry might be prevented.
